# Supplementary material for: EAE of Mice: Enzymatic Cross Site-Specific Hydrolysis of H2A Histone by IgGs against H2A, H1, H2B, H3, and H4 Histones and Myelin Basic Protein
Source: Int J Mol Sci. 2023 May 12;24(10):8636. doi: 10.3390/ijms24108636 (PMC10218518; doi:10.3390/ijms24108636)
Supplement: Supplementary file 1 [file ijms-24-08636-s001.zip › ijms-2276957-supplementary.pdf]

Supplementary Data

All methods and Figures on the changes in various parameters characterizing spontaneous and MOG-induced development of experimental autoimmune encephalomyelitis in EAE prone C57BL/6 male mice are taken from [1-3].

1. Doronin, V.B.; Parkhomenko, T.A.; Korablev, A.; et. al. Changes in different parameters, lymphocyte proliferation and hematopoietic progenitor colony formation in EAE mice treated with myelin oligodendrocyte glycoprotein. J Cell Mol Med. 2016; 20: 81–94.
2. Doronin, V.B.; Korablev, A.; Toporkova, L.B.; Aulova, K.S.; et al. Changes in several disease parameters including abzymes and hematopoietic progenitor colony formation in brain inflammation and demyelination. *J. Neurol. Neurol. Disord.* **2017**, *3*, 302.
3. Aulova, K.S.; Toporkova, L.B.; Lopatnikova, J.A.; Alshevskaya, A.A.; et al. Changes in cell differentiation and proliferation lead to production of abzymes in EAE mice treated with DNA-Histone complexes. *J. Cell Mol. Med.* **2018**, *22*, 5816-32.

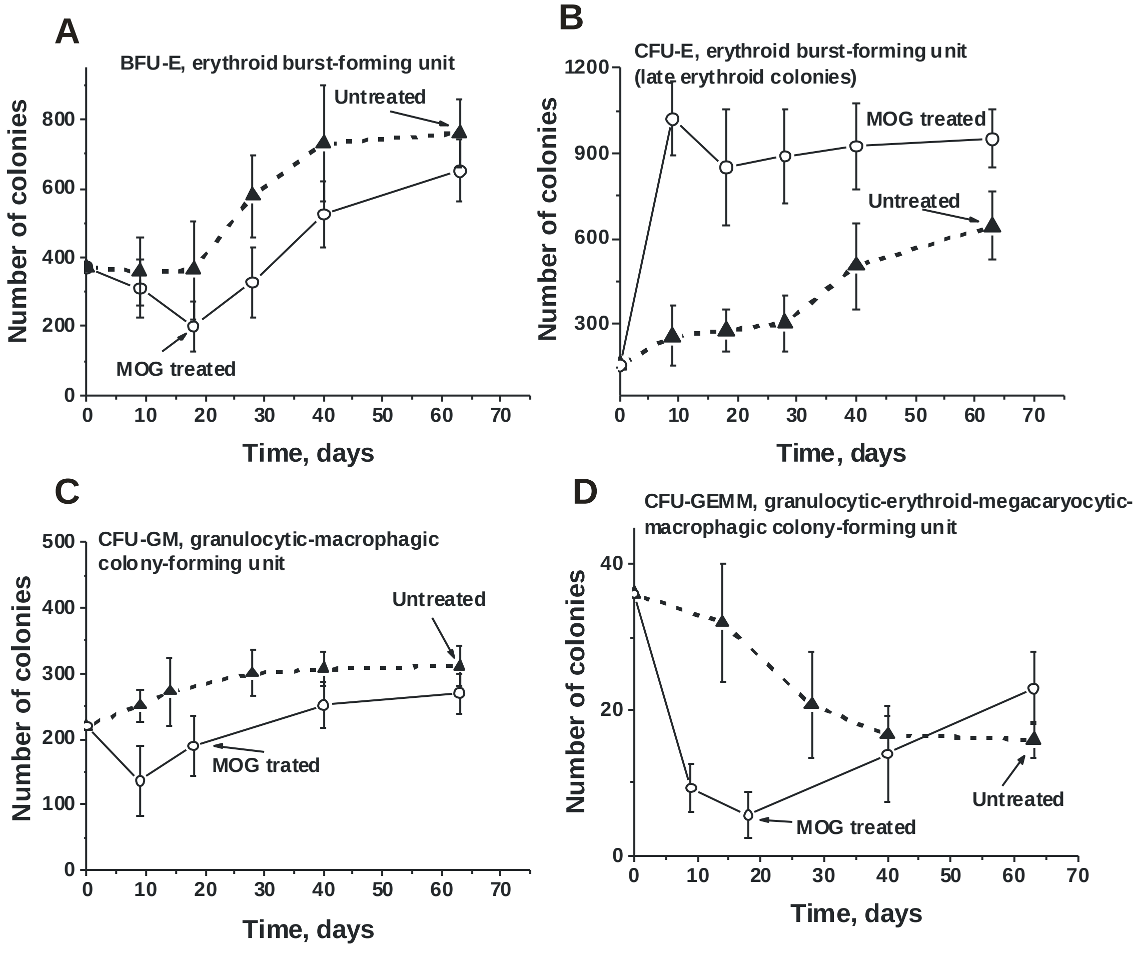

Supplementary Figure S1. In time changes of an average relative content of different colony-forming units of bone marrow progenitor colonies in the case of untreated and MOG-treated male C57BL/6 mice; average number of the colonies corresponding to 7 mice of each group is given and types of progenitor colonies (BFU-E (A), CFU-E (B), CFU-GM (C), and CFU-GEMM (D) are shown [1-3].

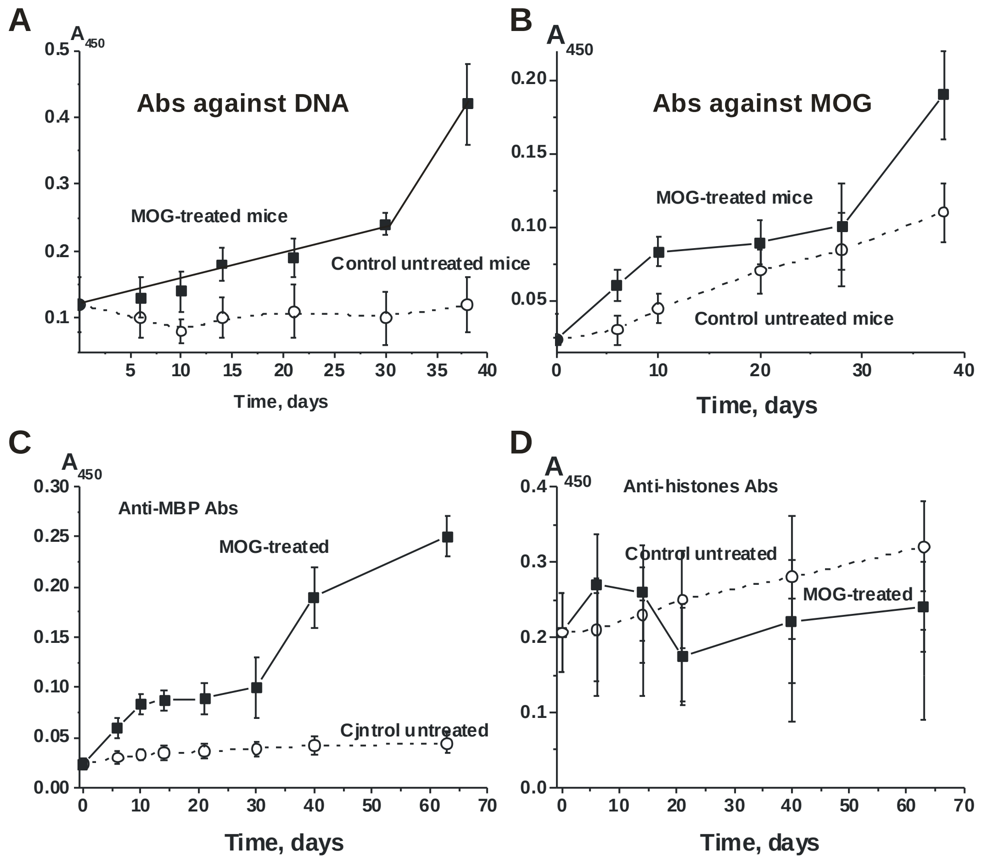

Supplementary Figure S2 The in-time changes in average values of anti-DNA (A), anti-MOG (B), anti-MBP (C), and anti-histones (D) Abs concentration in C57BL/6 male mice before and after their immunization with MOG [1-3].

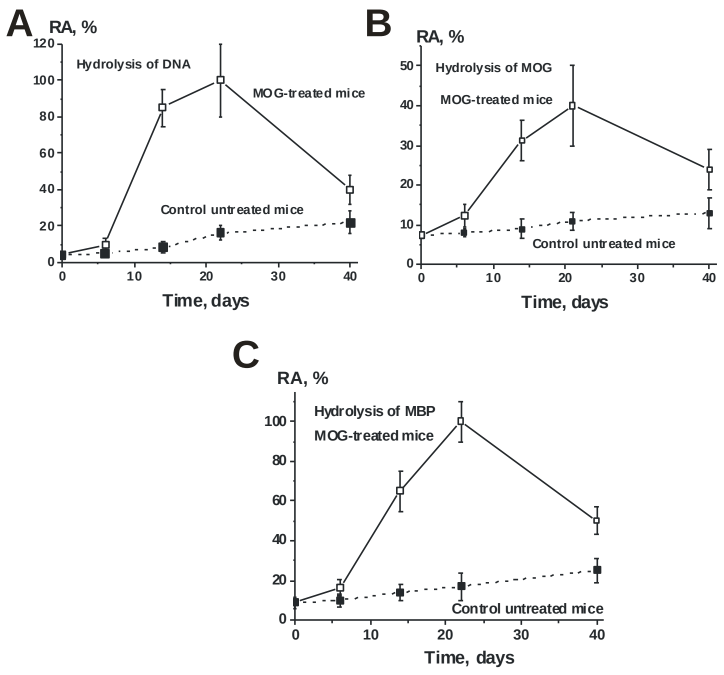

Supplementary Figure S3 The in-time changes in average relative activities (RA) in the hydrolysis by IgGs from sera of C57BL/6 male mice of DNA (A), MOG (B), and MBP (C) before and after mice immunization with MOG [1-3].

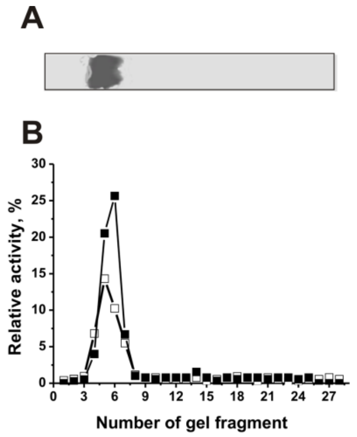

SupplementaryFigure S4. The IgG_{mix} (14 μg) homogeneity analysis by SDS-PAGE under non-reducing conditions in the absence of DTT (**A**); silver staining. Panel **A** demonstrates the position of IgGs. The relative activities (RA, %) in the hydrolysis of five histones (■) and MBP (□) were estimated using eluates of gel fragments (2-3 mm) (**B**). After incubation for 24 h with eluates, complete hydrolysis of all substrates was taken for 100% (**B**). The errors of the relative activities estimation from two independent experiments did not exceed 7–10%.
